# Pioglitazone on nonalcoholic steatohepatitis: A systematic review and meta-analysis of 15 RCTs

**DOI:** 10.1097/MD.0000000000031508

**Published:** 2022-11-18

**Authors:** Yan Zhao, Wenli Zhao, Hongwu Wang, Ye Zhao, Huaien Bu, Hirokazu Takahashi

**Affiliations:** a Department of Public Health, International College, Krirk University, Bangkok, Thailand; b Graduate School, Tianjin University of Traditional Chinese Medicine, Tianjin, China; c Department of Public Health, International College, Krirk University, Bangkok, Thailand; d Liver Center, Saga University Hospital, Saga University, Saga, Japan; e School of Health science and Engineering, Tianjin University of Traditional Chinese Medicine, Tianjin, China; f Department of Public Health, International College, Krirk University, Bangkok, Thailand; g School of Health Science and Engineering, Tianjin University of Traditional Chinese Medicine, Tianjin, China; h Liver Center, Saga University Hospital, Faculty of Medicine, Saga University, Saga, Japan.

**Keywords:** meta-analysis, nonalcoholic steatohepatitis, pioglitazone, randomized controlled trial, systematic review

## Abstract

Nonalcoholic steatohepatitis is regarded as a risk factor of many liver diseases.

**Methods:**

Relevant studies were searched from The National Library of Medicine, Cochrane Library, Elsevier, China National Knowledge Infrastructure, Web of Science and WANFANG databases. A total of 15 eligible studies were analyzed in the Reviewer Manager 5.3 software, including 7 English articles and 8 Chinese articles.

**Results:**

Fifteen studies are selected for this meta-analysis, which includes totally 623 patients in the treatment group and 594 patients in the control group. As a result, 8 studies show that the total effective rate of the treatment group is higher than that of the control group [*Z* = 3.64, 95% confidence intervals (CI): 1.78 (1.31–2.43), *P* = .0003]; eleven studies show that fasting plasma glucose levels of the experimental group are lower than that of the control group [*Z* = 4.38, 95% CI: −0.95 (−1.38 to −0.53), *P* < .0001]; ten studies show that glutamic-pyruvic transaminase levels of the experimental group are lower than that of the control group [*Z* = 3.69, 95% CI: −11.76 (−18.01 to −5.51), *P* = .0002]; 6 studies show that glutamic oxalacetic transaminase levels of the experimental group are lower than that of the control group [*Z* = 7.40, 95% CI: −3.01 (−3.81 to −2.22), *P* < .00001]; 6 studies show that gamma-glutamyl transpeptidase levels of the experimental group are lower than that of the control group [*Z* = 2.43, 95% CI: −23.77 (−42.98 to −4.57), *P* = .02]; 9 studies show that triglyceride levels of the experimental group are lower than that of the control group [*Z* = 3.06, 95% CI: −0.62 (−1.01 to −0.22), *P* = .002]; 6 studies show that the homeostasis model assessment of insulin resistance of the experimental group is lower than that of the control group [*Z* = 3.22, 95% CI: −2.33 (−3.75 to −0.91), *P* = .001]; 6 studies show that the glycated hemoglobin A1c of the experimental group is lower than that of the control group [*Z* = 4.50, 95% CI: −1.90 (−2.72 to −1.07), *P* < .00001]; five studies show that the fasting insulin of the experimental group is lower than that of the control group [*Z* = 3.42, 95% CI: −2.25 (−3.53 to −0.96), *P* = .0006].

**Conclusion:**

Pioglitazone intake is effective in nonalcoholic steatohepatitis management.

## 1. Introduction

Nonalcoholic steatohepatitis (NASH) affects about 3% to 6% of adults and its prevalence is increasing.^[1]^ NASH is a major cause of chronic liver disease. It is closely related to obesity, dyslipidemia, diabetes and metabolic syndrome. In a small number of patients with NASH, the disease may progress and eventually lead to advanced fibrosis, cirrhosis and hepatocellular carcinoma.^[2]^ Most NASH patients have no symptom or nonspecific symptom, most commonly, patients with NASH were identified after examination for unrelated conditions.^[1]^ According to current studies, NASH is an inflammatory subtype of nonalcoholic fatty liver disease (NAFLD), which is also the most serious form. It is characterized by inflammatory infiltration, hepatocyte damage and excessive fat accumulation, with or without fibrosis.^[3]^ However, the exact pathogenesis of NASH is still unclear. At present, we know the following aspects. First, high fat diet plays an important role in the pathogenesis of NAFLD. Lipid in diet, lipolysis of visceral fat and production of new fat have different contributions to lipid storage in liver; Lipolysis and new adipogenesis are strictly controlled by many hepatocyte nuclear receptors, which account for the largest proportion in the free fatty acids (FFAs) pool of the liver. Liver FFAs are partially oxidized as energy source, partially stored as triglyceride (TG), and finally as very low-density lipoprotein module. However, the whole process can’t be done to dispose of excess fat and TGs will accumulate in the liver.^[4]^ Secondly, Insulin resistance is 1 of the key factors in the occurrence of NASH, which leads to the increase of liver new adipogenesis and the weakening of the inhibition of adipose tissue decomposing fat, thus increasing the flow of FFAs to the liver.^[5]^ Insulin resistance can also promote adipose tissue dysfunction, thereby changing the production and secretion of adipokine n and inflammatory cytokines.^[6]^ Thirdly, fat mainly accumulates in the liver in the form of triglycerides, but triglycerides themselves are not toxic. Therefore, the grade or severity of steatosis cannot predict liver injury, inflammation or fibroma.^[7]^ Lipotoxicity refers to the imbalance of lipid environment and (or) intracellular lipid composition, resulting in the accumulation of harmful lipids, which may be related to organelle dysfunction, cell injury and death. Increased lipotoxicity occurs simultaneously from high levels of FFAs, free cholesterol and other lipid metabolites, therefore, mitochondrial dysfunction is activated by oxidative stress, reactive oxygen species and endoplasmic reticulum stress.^[8]^ Fourthly, a large number of hepatocyte cytochrome enzymes are found in mitochondria, which participate in hepatocyte injury and develop into NASH by promoting oxidative stress, inflammation, protein modification and insulin resistance. Hepatocyte cytochrome enzymes hydrolyzes various small molecules, such as FFAs and ethanol, into byproducts (toxic superoxide anion), which changes the respiratory chain of mitochondria and destroys mitochondrial components.^[9]^ Fifthly, endoplasmic reticulum is an intracellular organelle, most of which secrete and membrane proteins are folded, and are sensitive to FFAs. This leads to the accumulation of unfolded or misfolded proteins. This accumulation activates the unfolded protein response to reconstruct homeostasis in the body; if this response fails, other pressure sensor proteins. For example, inositol requires enzyme 1, activating transcription factor 6 and protein kinase receptor like endoplasmic reticulum kinase (perk), which triggers autophagy.^[10,11]^ Inositol requires enzyme 1 splices and activates transcription factor X-box binding protein 1.^[12]^ X-box binding protein 1 interacts with various inflammatory cascades by activating the amino-terminal kinase and inhibitor of nuclear factor kappa B kinase-nuclear factor kappa B signal transduction and reactive oxygen species production.^[13]^ Lastly, the mechanism of NASH is related to intestinal barrier dysfunction, which may increase bacterial translocation and lead to liver inflammation. Indeed, the level of circulating endotoxin in NASH patients is higher than that of healthy individuals.^[14]^ Endotoxin can induce inflammatory reaction by activating hepatitis cells.^[15]^ According to studies published in 2015, CX3C chemokine receptor 1 plays a role in controlling intestinal barrier permeability. Deletion of CX3C chemokine receptor 1 has a negative effect on intestinal barrier function and aggravates steatohepatitis in NASH mice.^[16]^ Some findings suggest that the intestinal barrier dysfunction increases liver permeability and bacterial translocation, leading to NASH induction.^[17]^ Therefore, the pathogenesis of NASH maybe caused by multiple factors, and more research is needed to solve the mystery.

There are also many statements about its treatment. At present, there is no recognized drug therapy, and the progress of treatment is slow.^[18]^ Dietary and lifestyle changes are now the primary treatment for patients with NASH.^[19]^ Mediterranean diet can effectively reduce liver fat, even if not lose weight, is the most recommended diet. It is characterized by a reduction in carbohydrate intake, especially sugar and refined carbohydrates, and an increase in monounsaturated fatty acids and omega-3 fatty acids.^[20]^ Experimental studies show that a diet rich in omega-3 polyunsaturated fatty acids can improve insulin sensitivity,^[21]^ it can reduce the content of triglyceride in liver and improve steatohepatitis.^[22]^ Monounsaturated fatty acids have a good effect on blood lipid level.^[23]^ Exercise also has recognized benefits in improving overall cardiovascular health, which is the leading cause of death in NAFLD patients. These benefits, including improved liver and peripheral insulin resistance, may not be associated with weight loss.^[24]^ One study shows that the group with high exercise intensity (>250 min/wk) has favorable changes in metabolic parameters and a significant decrease in liver fat content compared with those less than 250 min/wk.^[25]^ Bariatric surgery is suitable for patients with severe or morbid obesity, and a variety of operations have been performed. Most patients undergoing bariatric surgery also have NAFLD. It is well known that weight loss can improve insulin sensitivity and play a beneficial role in reducing visceral fat. A new study reports that bariatric surgery can improve body mass index (BMI), insulin resistance index and other markers significantly, and NASH disappeared in 85% of patients.^[26]^

Although the above general treatments have obvious effect, there is no FDA approval for specific drugs for NASH. However, the pioglitazone, a thiazolidinedione insulin sensitizer through peroxisome proliferator activated receptor (PPAR-γ), has shown some benefit in some randomized controlled trials.^[1]^ Studies show that the pioglitazone can improve insulin and glucose parameters, increase lipid storage in the subcutaneous adipose tissue, increase the adiponectin, and reduce the lipid toxicity of liver.^[27]^ However, there are still some problems worthy of attention in the clinical application of pioglitazone. Increased risk of prostate or pancreatic cancer, weight gain, fluid retention, female fracture and increased cardiovascular events.^[18]^ Therefore, pioglitazone in the treatment of NASH is still controversial, which needs to be further clarified. We performed a meta-analysis to investigate the relationship between pioglitazone and NASH.

## 2. Methods

### 2.1. Research strategy

The National Library of Medicine, Cochrane Library, Elsevier, China National Knowledge Infrastructure, Web of Science and WANFANG databases were searched from their earliest records to November 2021 using the following key words: NASH and pioglitazone. The search was performed by combining the search terms with the subject words.

### 2.2. Inclusive criteria

The inclusion criteria are randomized controlled clinical trials. The treatment group is treated with pioglitazone alone or on the basis of conventional treatments, while the control group is treated with placebo or conventional treatment (including diet, exercise, etc.) for NASH. Trials investigating the impact of pioglitazone on at least one outcome of glutamic-pyruvic transaminase (ALT), glutamic oxalacetic transaminase (AST), BMI, weight, fasting plasma glucose (FPG), gamma-glutamyl transpeptidase (GGT), homeostasis model assessment of insulin resistance (HOMA-IR), glycated hemoglobin A1c (HbA1c), fasting insulin (FNS), fibrosis and histological improvements are considered for inclusion.

### 2.3. Exclusion criteria

We exclude repetitive articles; nonintervention studies such as case-control studies, case reports and experiences, theoretical studies and reviews; and nonclinical trials, such as animal tests.

### 2.4. Quality evaluation and data extraction

The methodological quality of the included studies is evaluated based on the quality assessment criteria recommended in the Cochrane systematic review manual. The main evaluation criteria include the following: a randomly assigned method, allocation concealment, use of blinding, data integrity, selectively reported results, and the presence of bias (“low risk” indicates a low risk of bias; “high risk” indicates a high risk of bias; and “unclear risk” indicates that the literature does not provide sufficient information for bias assessment). The data quality was evaluated by 2 independent researchers. Inconsistent opinions were resolved via a discussion or by soliciting the advice of a third party regarding the inclusion of a particular study.

### 2.5. Statistical analysis

All statistical analyses were performed using Review Manager (version5.3). The risk ratio is used for count data, while the standardized mean difference (SMD) is adopted for continuous variables as effect size. Respectively, both are demonstrated with the effect size and 95% confidence intervals (CI). If there is no heterogeneity among the studies, that is, a *P*-value greater than .10 or *I*^2^ less than 50%. It is explained that the heterogeneity of the research is small, and the fixed effect model is used to analyze. A *P*-value less than .10 or *I*^2^ greater than 50% suggested that there is obvious heterogeneity among the included studies, and the random effect model is used to combine the effect volume. The bias of the study is analyzed by funnel plot.

## 3. Results

### 3.1. Study selection

**Figure 1.**
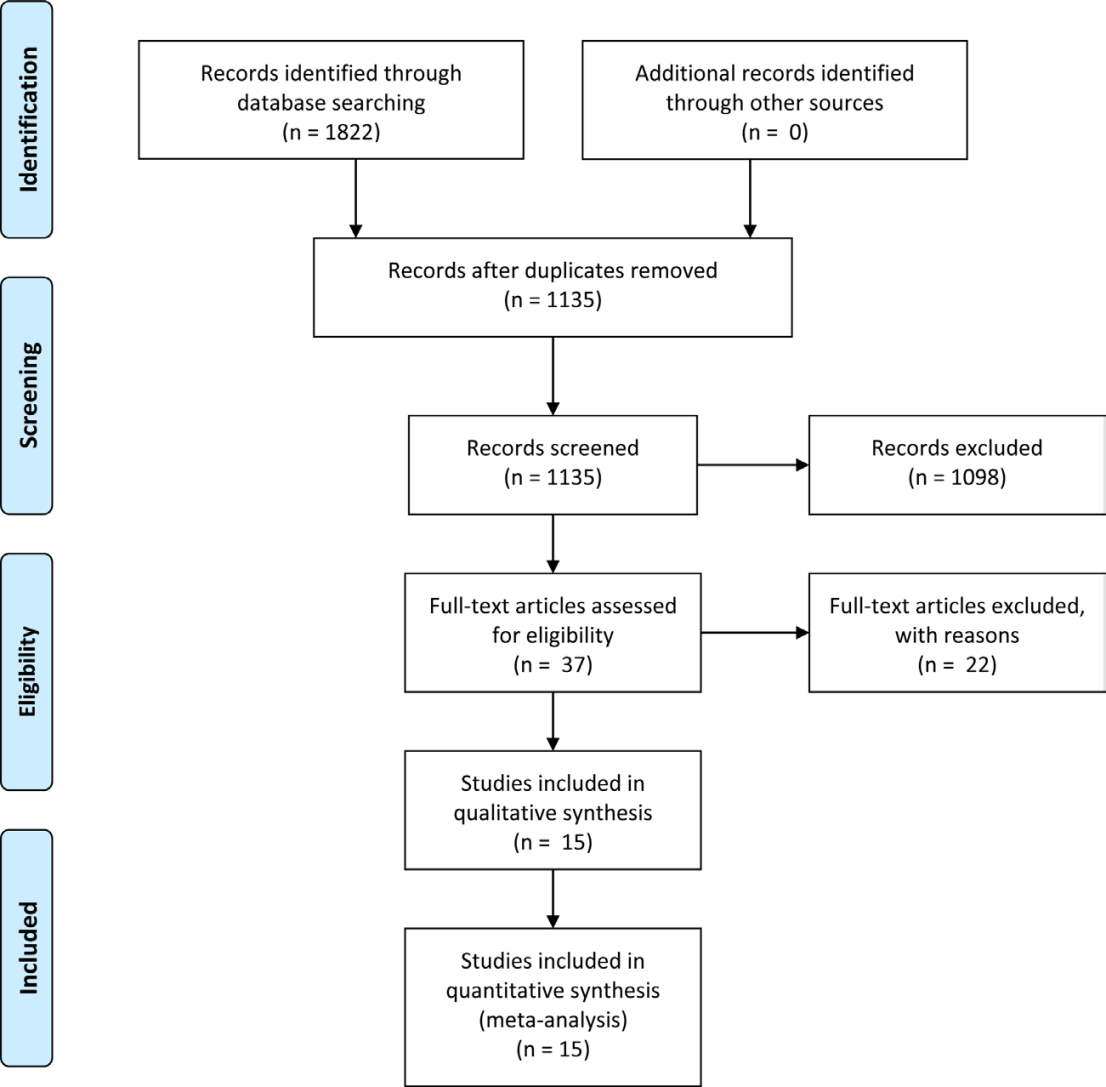
PRISMA 2009 flow diagram.

A total of 1822 articles are searched from English and Chinese databases. 1135 of which are duplicated and 1120 are generally excluded based on the inclusion criteria. Finally, 15 eligible articles are included in the meta-analysis. The study selection procedure is outlined in Figure 1.

### 3.2. Study characteristics and quality

The study information is shown in Table 1, and the quality of the study evaluation is shown in Table 2.

**Table 1:** Study characteristics.

Author	Year	Age	M/F	Duration (wk)	Treatment		Control		Evaluation indicator
					Cases	Pioglitazone	Cases	Measures	
Sanyal^[28]^	2010	T:47.0 ± 12.6 C:45.4 ± 11.2	68/95	96	80	30 mg/d	83	Placebo	ALT, AST, weight, TG, FPG, BMI, GGT, total effective rate
Gastaldelli^[29]^	2010	49-57	-	24	26	45 mg/d	21	Placebo	Plasma adiponectin, insulinsensitivity
Aithal^[30]^	2008	27-73	45/29	48	37	30 mg/d	37	Placebo	Weight, ALT, HbA1c, FPG, GGT
Sanchez^[31]^	2019	51 ± 1	55/23	72	39	45m g/d	39	Placebo	FPG, HbA1c, HOMA-IR Fasting plasma insulin
Cusi^[32]^	2016	T:52 ± 10 C:49 ± 11	71/30	72	51	45 mg/d	50	Placebo	Weight, BMI, ALT, AST, FPG, TG, fasting plasma insulin, total effective rate
Balas^[33]^	2007	T:51 ± 1.6 C:48.4 ± 3.1	16/19	24	21	45 mg/d	14	Placebo	HbA1c, weight, BMI
Belfort^[34]^	2006	T:51 ± 7 C:51 ± 10	21/26	24	26	45 mg/d	21	Placebo	Weight, BMI, ALT, AST, FPG, TG, fasting plasma insulin
Xiang^[35]^	2007	T:44.5 ± 27 C:43.5 ± 20	27/16	24	22	15 mg/d	21	Placebo	ALT, GGT, TG, HOMA-IR
Chen^[36]^	2018	T:39.4 ± 10.2 C:38.6 ± 9.4	72/48	24	60	15 mg/d + RT	60	RT	Total effective rate
Guo^[37]^	2014	45.0 ± 6.2	42/38	12	40	15 mg/d + RT	40	RT	FPG, BMI, ALT, AST, HOMA-IR, TG, total effective rate
He^[38]^	2008	40.0 ± 6.0	49/37	16	46	30 mg/d + RT	40	RT	FPG, HbA1c, TG, HOMA-IR
Jin^[39]^	2010	T:50.3 ± 12.8 C:53.7 ± 10.1	65/55	24	60	30 mg/d + RT	60	RT	ALT, AST, GGT, FPG, FNS Total effective rate
Li^[40]^	2014	67.1 ± 15.6	-	52	60	15 mg/d + RT	56	RT	FNS, BMI, FPG, HbA1c, TG, ALT, AST, HOMA-IR, total effective rate
Xiang^[41]^	2009	T:48.0 ± 5.7 C:47.3 ± 6.1	23/20	12	23	15 mg/d + RT	20	RT	FPG, HbA1C, TG, ALT Total effective rate
Xie^[42]^	2014	T:53.4 ± 10.3 C:50.3 ± 10.5	35/29	24	32	30 mg/d + RT	32	RT	BMI, ALT, GGT, FPG, TG, FNS HOMA-IR, total effective rate

**Table 2:** Study quality evaluation.

Included studies	Random allocation	Allocation concealment	Double blind method	Evaluation of blindness	Data integrity	Selective report	others
Sanyal 2010	Unclear	Low risk	Unclear	Low risk	Low risk	Low risk	Unclear
Gasaldelli 2010	Unclear	Unclear	High risk	Low risk	Low risk	Low risk	Low risk
Aithal2008	Low risk	Low risk	Lowrisk	Low risk	Low risk	Low risk	Unclear
Sanchez 2019	Low risk	Unclear	Low risk	Low risk	Unclear	Low risk	Low risk
Cusi 2016	Low risk	Low risk	Low risk	Low risk	Low risk	Low risk	Low risk
Balas 2007	Unclear	Unclear	Unclear	Low risk	Low risk	Unclear	Unclear
Belfort 2006	Unclear	Low risk	Low risk	Unclear	Low risk	Low risk	Low risk
Xiang 2007	Unclear	Unclear	Unclear	High risk	Low risk	Low risk	Unclear
Chen 2018	Low risk	Unclear	Unclear	Unclear	Low risk	Unclear	Unclear
Guo 2014	Unclear	Unclear	Unclear	Unclear	Low risk	Low risk	Unclear
He 2008	Unclear	Unclear	Unclear	Unclear	Unclear	Unclear	Unclear
Jin 2010	Low risk	Unclear	Unclear	Low risk	Low risk	Low risk	Unclear
Li2014	Unclear	Unclear	Unclear	Unclear	Low risk	Low risk	Low risk
Xiang 2009	Unclear	Unclear	Unclear	Unclear	Low risk	Low risk	Unclear
Xie 2014	Unclear	Unclear	Unclear	Unclear	Low risk	Unclear	Low risk

### 3.3. Meta-analysis of outcome

#### 3.3.1. Total effective rate.

**Figure 2.**
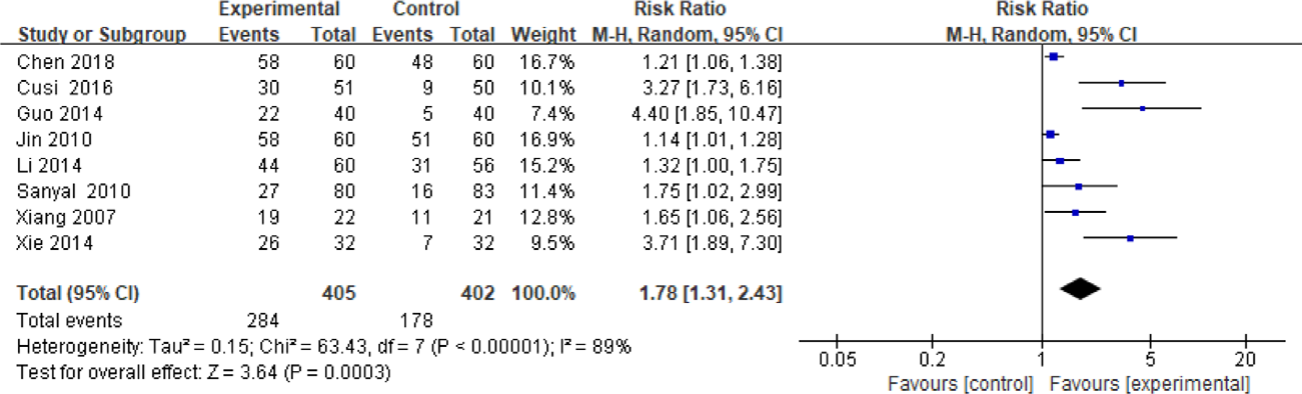
Changes in total effective rate of the experimental group compared with the control group.

In general, 8 studies use the total effective rate as an indicator of the effectiveness of pioglitazone-guided interventions. The results are shown in Figure 2. A total of 462 patients with NASH are included in this evaluation (284 in the experimental group and 178 in the control group). The heterogeneity test shows that the heterogeneity is large (*I*^2^ = 0.89), so the random effect model is used. The results show that the difference is significant, the effective rate of the experimental group is 78% higher than that of the control group [risk ratio = 1.78, 95% CI: (1.31–2.43)].

#### 3.3.2. Weight.

**Figure 3.**
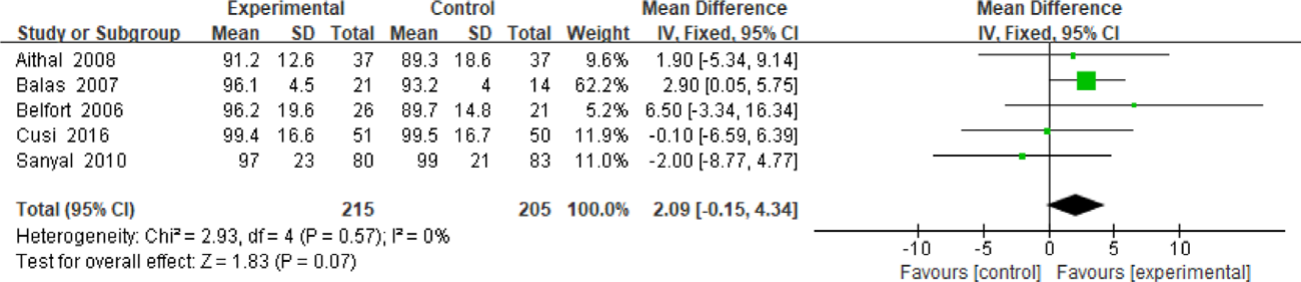
The experimental group compared with the control group in weight changes after treatment.

Five studies use the weight as an indicator of the effectiveness of pioglitazone-guided interventions. A total of 420 patients with NASH are included in this evaluation (215 in the experimental group and 205 in the control group), according to the results of meta-analysis (*P* = .57, *I*^2^ = 0%). No heterogeneity is found between the studies, that is why the fixed effect model is used to calculate. There is no significant difference in changes in weight between the experimental group and the control group. The results are shown in Figure 3.

#### 3.3.3. Body mass index.

**Figure 4.**
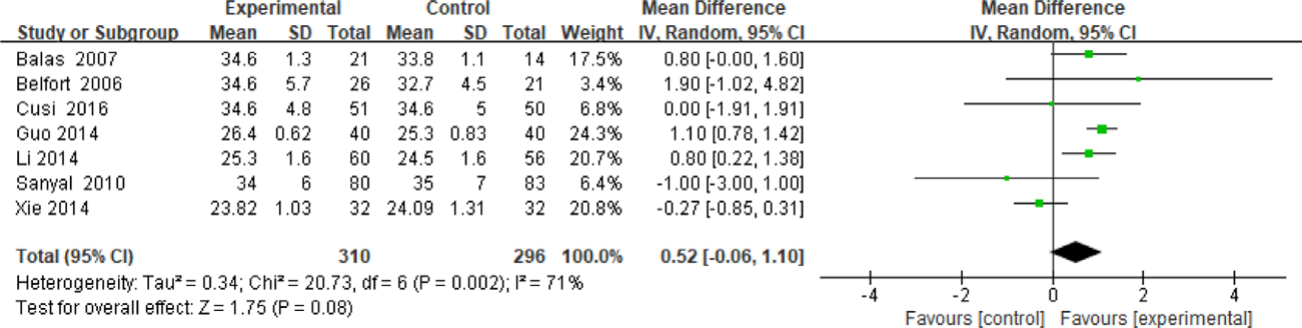
The experimental group compared with the control group in BMI changes after treatment. BMI = body mass index.

Seven studies use BMI as an indicator of the effectiveness of pioglitazone-guided interventions. A total of 606 patients with NASH are included in this evaluation (310 in the experimental group and 296 in the control group). The heterogeneity was large (*P* = .002, *I*^2^ = 0.71), so the random effects model is used. Changes in BMI between the experimental group and the control group are no significant, as shown in Figure 4.

#### 3.3.4. FPG level.

**Figure 5.**
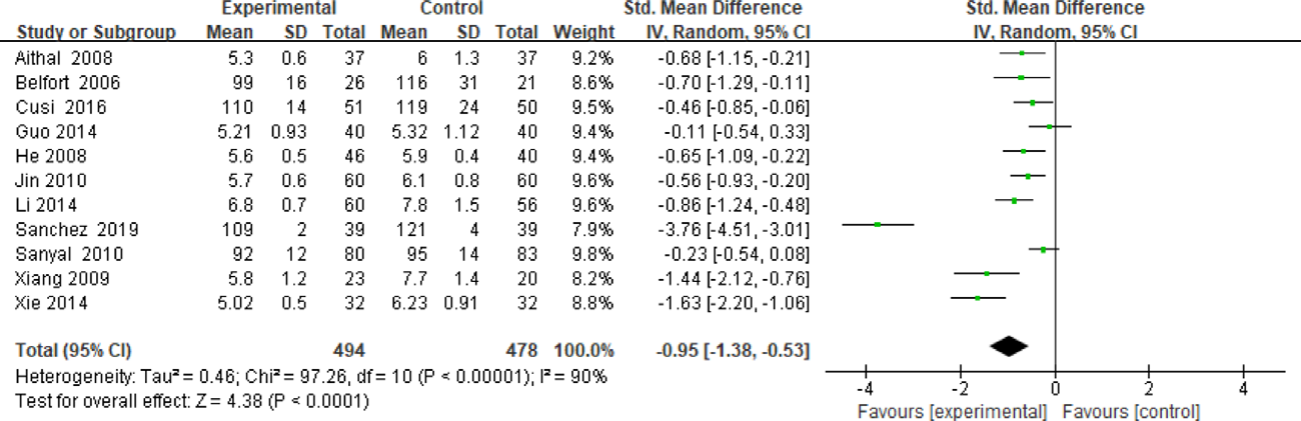
The experimental group compared with the control group in FPG changes after treatment. FPG = fasting plasma glucose.

Eleven studies use the FPG level as an indicator of the effectiveness of pioglitazone-guided interventions. A total of 972 patients with NASH are included in this evaluation (494 in the experimental group and 478 in the control group). The heterogeneity is large (*P* < .00001, *I*^2^ = 0.90). So, the random effect model is used. The meta-analysis shows that the FPG level of NASH patients treated with pioglitazone are lower than those received the placebo or conventional treatments [SMD = −0.95, 95% CI: (−1.38 to −0.53)], as shown in Figure 5.

#### 3.3.5. ALT level.

**Figure 6.**
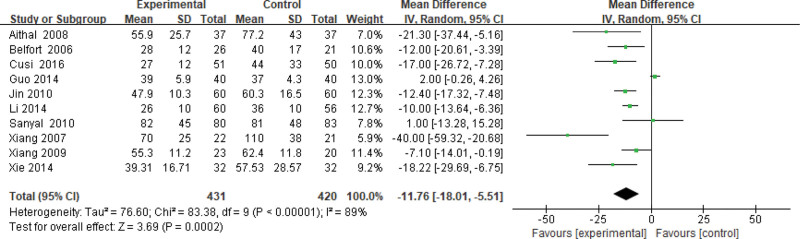
The experimental group compared with the control group in ALT changes after treatment. ALT = glutamic-pyruvic transaminase.

Ten studies use the ALT level as an indicator of the effectiveness of pioglitazone-guided interventions. A total of 851 patients with NASH are included in this evaluation (431 in the experimental group and 420 in the control group). The heterogeneity is large (*P* < .00001, *I*^2^ = 0.89). So, the random effect model is used. Changes in the ALT level between the experimental group and the control group are significant. The ALT level of NASH patients treated with pioglitazone are lower than those received the placebo or conventional treatments [*Z* = 3.69, *P* = .0002, MD = −11.76, 95% CI: (−18.01 to −5.51)], as shown in Figure 6.

#### 3.3.6. AST level.

**Figure 7.**
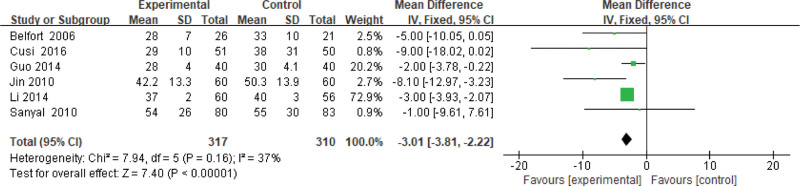
The experimental group compared with the control group in AST changes after treatment. AST = glutamic oxalacetic transaminase.

Six studies use the AST level as an indicator of the effectiveness of pioglitazone-guided interventions. A total of 627 patients with NASH are included in this evaluation (317 in the experimental group and 310 in the control group). The heterogeneity is small (*P* = .16, *I*^2^ = 0.37). So, the fixed effect model is used. The meta-analysis shows that the AST level of NASH patients treated with pioglitazone are lower than those received the placebo or conventional treatments [*Z* = 7.40, *P* < .0001, MD = −3.01, 95% CI: (−3.81 to −2.22)], as shown in Figure 7.

#### 3.3.7. GGT level.

**Figure 8.**
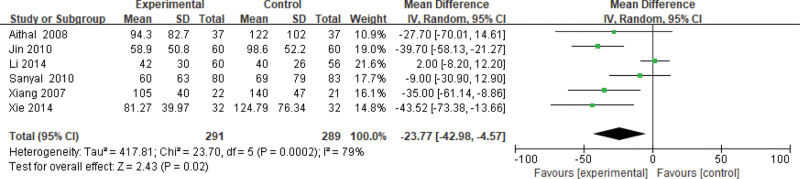
The experimental group compared with the control group in GGT changes after treatment. GGT = gamma-glutamyl transpeptidase.

Six studies use the GGT level as an indicator of the effectiveness of pioglitazone-guided interventions. A total of 580 patients with NASH are included in this evaluation (291 in the experimental group and 289 in the control group). The heterogeneity is large (*P* = .0002, *I*^2^ = 0.79). So, the random effect model is used. Changes in the GGT level between the experimental group and the control group are significant. The GGT level of NASH patients treated with pioglitazone are lower than those received the placebo or conventional treatments [*Z* = 2.43, *P* = .02, MD = −23.77, 95% CI: (−42.98 to −4.57)], as shown in Figure 8.

#### 3.3.8. TG level.

**Figure 9.**
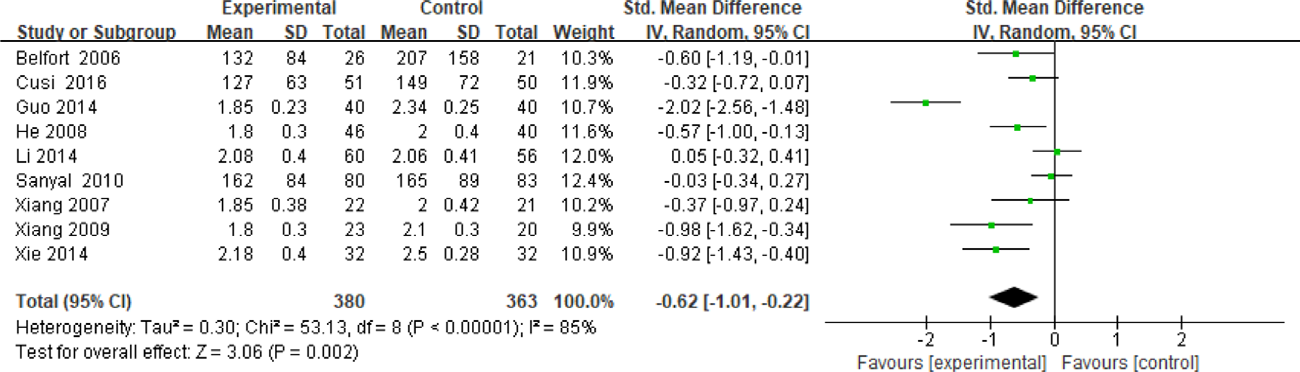
The experimental group compared with the control group in TG changes after treatment. TG = triglyceride.

Nine studies use the TG level as an indicator of the effectiveness of pioglitazone-guided interventions. A total of 743 patients with NASH are included in this evaluation (380 in the experimental group and 363 in the control group). The heterogeneity is large (*P* < .00001, *I*^2^ = 0.85). So, the random effect model is used. The meta-analysis shows that the TG level of NASH patients treated with pioglitazone are lower than those received the placebo or conventional treatments [*Z* = 3.06, *P* = .092, MD = −0.62, 95% CI: (−1.01 to −0.22)], as shown in Figure 9.

#### 3.3.9. HOMA-IR.

**Figure 10.**
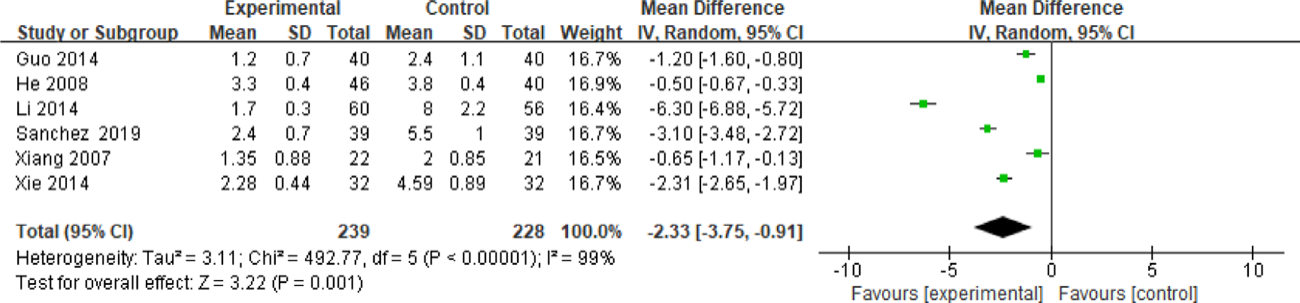
The experimental group compared with the control group in HOMA-IR changes after treatment. HOMA-IR = homeostasis model assessment of insulin resistance.

Six studies use the HOMA-IR as an indicator of the effectiveness of pioglitazone-guided interventions. A total of 467 patients with NASH are included in this evaluation (239 in the experimental group and 228 in the control group). The heterogeneity is large (*P* < .00001, *I*^2^ = 0.99). So, the random effect model is used. Changes in the HOMA-IR between the experimental group and the control group are significant. The HOMA-IR level of NASH patients treated with pioglitazone are lower than those received the placebo or conventional treatments [*Z* = 3.22, *P* = .001, MD = −2.33, 95% CI: (−3.75 to −0.91)], as shown in Figure 10.

#### 3.3.10. HbA1c level.

**Figure 11.**
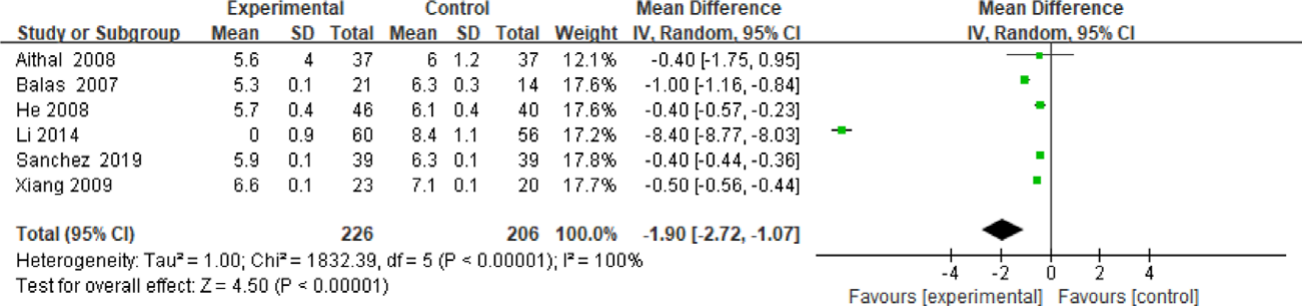
The experimental group compared with the control group in HbA1c changes after treatment. HbA1c = glycated hemoglobin A1c.

Six studies use the HbA1c level as an indicator of the effectiveness of pioglitazone-guided interventions. A total of 432 patients with NASH are included in this evaluation (226 in the experimental group and 206 in the control group). The heterogeneity is large (*P* < .00001, *I*^2^ = 1.00). So, the random effect model is used. Changes in the HbA1c level between the experimental group and the control group are significant. The HbA1c level of NASH patients treated with pioglitazone are lower than those received the placebo or conventional treatments [*Z* = 4.50, *P* < .00001, MD = −1.90, 95% CI: (−2.72 to −1.07)], as shown in Figure 11.

#### 3.3.11. FNS level.

**Figure 12.**
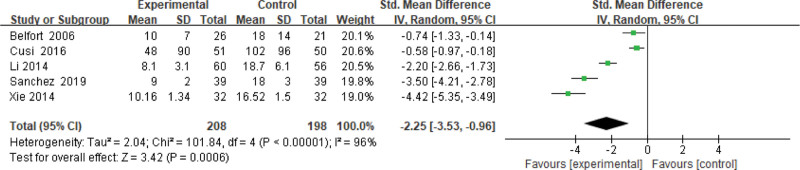
The experimental group compared with the control group in FNS changes after treatment. FNS = fasting insulin.

Five studies use the FNS level as an indicator of the effectiveness of pioglitazone-guided interventions. A total of 406 patients with NASH are included in this evaluation (208 in the experimental group and 1987 in the control group). The heterogeneity is large (*P* < .00001, *I*^2^ = 0.96). So, the random effect model is used. Changes in the FNS level between the experimental group and the control group are significant. The FNS level of NASH patients treated with pioglitazone are lower than those received the placebo or conventional treatments [*Z* = 3.42, *P* = .0006, SMD = −2.25, 95% CI: (−3.53 to −0.96)], as shown in Figure 12.

#### 3.3.12. Adverse reactions.

No major adverse reactions are reported in all studies, although some patients had adverse events (21 cases of lower extremity edema, 1 case of right upper abdominal pain, 1 case of weight gain, 1 case of rash, 1 case of vertigo, 1 case of diarrhea, 1 case of headache, 3 cases of mild liver enzyme elevation). After symptomatic medication, the symptoms disappeared automatically, which did not affect the follow-up study. However, its safety is still worthy of further study.

#### 3.3.13. Publication bias.

**Figure 13.**
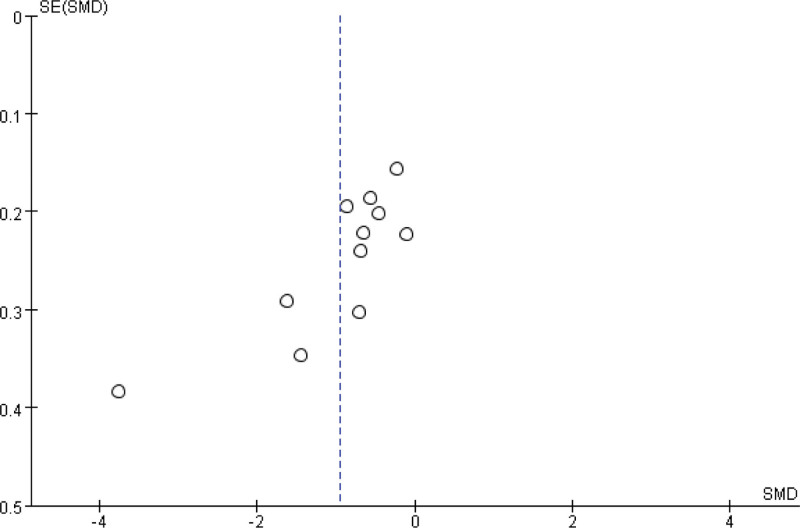
Funnel plot.

As shown in Figure 13, based on the FPG level, funnel plot is applied to evaluate the publication biases of all 15 studies. The results show no publication bias.

## 4. Discussion

### 4.1. Analysis of pioglitazone efficacy

In this meta-analysis, pioglitazone has a certain effect on patients with NASH. It can effectively improve the degree of NASH, liver function and blood glucose. Also, there is no major adverse events in the study. The change of each index comes from different mechanism. As demonstrated in the meta-analysis, the total effective rate of the experimental group for NASH patients rose by about 78% compared with that of the control group. The main reason may be that pioglitazone can improve the sensitivity of target tissue to insulin, reduce insulin resistance and regulate blood lipid.^[43]^ Most other parameters are lower in the experimental group than those in the control group. But there is no significant changing difference in weight or BMI. The main reason for the decline of FPG level may be that pioglitazone regulates the genes’ transcription related to insulin, so it may control the generation, transportation and utilization of the blood glucose. Pioglitazone downgrades fasting glucose by enhancing insulin-induced suppression of gluconeogenesis and glycogenolysis rather than by glucagon reduction.^[44]^ The main reason for the decline of HbA1c and TG level may be that pioglitazone can also increase uncoupling protein 1 expression in adipocytes and promote the energy consumption.^[45]^ Moreover, pioglitazone can significantly reduce ALT, AST, and GGT. All results indicate that pioglitazone can control the liver enzyme spectrum caused by fatty liver.

Considering the mechanism on reducing the steatosis and inflammation of liver, pioglitazone can promote the differentiation of white adipocytes, increase the number of small adipocytes and reduce the number of large adipocytes after activating PPAR γ in the body. Small adipocytes are more sensitive to insulin, which can promote glucose uptake, promote energy consumption and reduce the storage of excess energy in adipose tissue.^[46]^ A declining of FNS and HOMA-IR indicates that pioglitazone does not promote the secretion of islet β cells, however, it can increase the tissues’ insulin sensitivity. The main reason for the decline of FNS and HOMA-IR level may be that pioglitazone can down regulate the expression of tumor necrosis factor-α, leptin and resistin genes, and these cytokines are closely related to insulin resistance, which may be 1 of the mechanisms of pioglitazone enhancing insulin sensitivity.^[47]^

### 4.2. Limitations

Although the 15 articles included in this meta-analysis prove that pioglitazone is useful, there are still some limitations: Firstly, there are some differences in the condition and basic treatments of NASH among the studies, which is also the reason for the heterogeneity of some indicators. Secondly, only Chinese and English literatures are included, and other languages are not involved. Language restrictions may lead to inappropriate results. Thirdly, all clinical studies have small sample size which may affect the reliability of the analysis results. Finally, the RCTs included in this study are biased in research design, methodology and result reporting. The details provided, such as randomization method, allocation concealment and blind method, are insufficient. Therefore, the evidence strength of the results is affected.

### 4.3. Applications prospects

In recent years, NASH becomes a serious public health problem. Its symptoms and related complications seriously affect the quality of patients’ lives.^[48]^ Pioglitazone is an insulin sensitizer that selectively activates PPAR-γ.^[49]^ PPARs are the main regulators of genes related to the glucose metabolism and the fat metabolism.^[50]^ Pioglitazone can promote the uptake and the storage of fatty acids and up-regulate the expression of the insulin receptor substrate-1.^[51]^ It can reduce the level of serum fatty acids and improve the insulin sensitivity of liver, muscle and adipose tissue. So, pioglitazone can achieve the purpose of treating NASH.^[52]^ The liver damage caused by NASH is mainly manifested by the abnormal biochemical indexes of liver function. ALT, AST and GGT are commonly used in clinical practice to reflect the liver function.^[53]^ Among them, ALT mainly exists in mitochondria of hepatocytes, and the intracellular concentration is 1000 to 3000 times higher than that of serum.^[54]^ The concentration of AST in normal human serum is very low.^[55]^ GGT mainly exists in the intrahepatic bile duct epithelium and the cytoplasm of hepatocytes. When the intrahepatic and extrahepatic bile duct obstruction can lead to the increase of GGT in serum. When the liver lesions are serious, a large number of hepatocytes and serious damage, GGT will increase.^[56]^ In this meta-analysis, liver function indexes are significantly different before and after the pioglitazone treatment. ALT, AST and GGT are significantly decreased. However, some studies have shown that weight gain is common in patients taking thiazolidine 2 ketone drugs, which can cause fluid retention and congestive heart failure.^[57]^ In addition, studies on the effect of pioglitazone withdrawal also show a significant rebound in the ALT.^[58]^

## 5. Conclusion

Pioglitazone intake is effective in NASH management, including the total effective rate and other related clinical indexes. The treatment of NASH needs to be further verified.

## Acknowledgments

The authors thank Dr Bin Wang for assistance with data extraction.

## Author contributions

All authors contributed to the design and concept, performed the literature searches, wrote the manuscript and critiqued the successive versions, and approved the final manuscript. YZ coordinated the effort and integrated the sections and comments.

**Conceptualization:** Wenli Zhao, Huaien Bu.

**Data curation:** Yan Zhao.

**Formal analysis:** Yan Zhao, Ye Zhao.

**Funding acquisition:** Hongwu Wang.

**Investigation:** Yan Zhao, Ye Zhao.

**Methodology:** Wenli Zhao, Hongwu Wang, Ye Zhao, Hirokazu Takahashi.

**Project administration:** Hirokazu Takahashi.

**Resources:** Huaien Bu, Hirokazu Takahashi.

**Software:** Wenli Zhao.

**Supervision:** Huaien Bu, Hirokazu Takahashi.

**Validation:** Hirokazu Takahashi.

**Writing – original draft:** Huaien Bu.

**Writing – review & editing:** Huaien Bu.
